# Patients with genital ambiguity referred without a sex definition: the relationship between clinical picture and defined sex of rearing

**DOI:** 10.1016/j.jped.2024.05.001

**Published:** 2024-05-29

**Authors:** Henrique C. Santos, Beatriz A. Barros, Mayra S. El-Beck, Marcio L. Miranda, Mara S. Guaragna, Helena Fabbri-Scallet, Taís N. Mazzola, Tarsis P. Vieira, Maricilda P. Mello, Antonia P. Marques-de-Faria, Andrea T. Maciel-Guerra, Gil Guerra-Junior

**Affiliations:** Universidade Estadual de Campinas (UNICAMP), Faculdade de Ciências Médicas e Hospital das Clínicas Universitárias, Grupo Interdisciplinar para o Estudo da Determinação e Diferenciação do Sexo, Campinas, SP, Brazil

**Keywords:** Genital ambiguity, Sex of rearing, Karyotype, Etiology, Sex

## Abstract

**Objective:**

It was to verify the association between the definition of sex of rearing and, clinical and cytogenetic features among patients with genital ambiguity referred without a sex assignment.

**Methods:**

The sample consisted of 133 patients with genital ambiguity seen at a single reference service. These patients did not have a defined social sex at the first consultation and their etiological diagnosis was obtained during follow-up.

**Results:**

A total of 133 cases were included, 74 of which were reared as males and 59 as females. No correlation was found between the year of birth and the year of the first consultation with the definition of sex of rearing. However, the definition of sex of rearing was associated with age at the first consultation, severity of genital ambiguity, presence of palpable gonad(s), presence of uterus on ultrasound, karyotype, and diagnosis. Palpable gonad(s), more virilized genitalia, absence of a uterus on ultrasound, 46, XY karyotype, or a karyotype with sex chromosome abnormalities emerged as strong predictors for defining male sex. All 77 (58 %) patients over 18 years old had a gender identity in accordance with the sex of rearing; though 9 of 77 (12 %) had homo or bisexual orientation, especially girls with Congenital Adrenal Hyperplasia.

**Conclusions:**

Clinical and cytogenetic data were strongly associated with the definition of the sex of rearing of children with genital ambiguity referred to a DSD center without sex assignment. Management in a specialized center allows the establishment of a gender identity in accordance with the sex of rearing.

## Introduction

The 2006 Chicago Consensus proposed the term Disorder of Sex Development (DSD) to encompass any congenital disease in which the chromosomal, gonadal, or anatomical constitution was atypical.^1^ DSD can manifest as genital ambiguity at birth, absent, incomplete, or atypical puberty, or early gonadal failure or infertility in young adults.^1^^,^^2^ The Chicago Consensus also proposed the classification of DSDs based on karyotype.^1^

In most newborns, the definition of sex of rearing is carried out correctly and without any difficulty only by checking the characteristics of the external genitalia. However, in cases of DSD, the definition of sex of rearing can only be achieved when other data are considered.^1^

The definition of sex of rearing depends on the concordance of the appearance of external genitalia (external genital sex) with internal genitalia (internal genital sex) and gonads (gonadal sex). In general, a 46,XY karyotype leads to the development of testes in individuals with internal and external male genitalia, whereas a 46,XX karyotype will result in the development of ovaries, with the presence of internal and external female genitalia. Hormonal differentiation (endocrinological sex) cannot be underestimated as it plays a basic role not only during puberty when individuals develop secondary sexual characteristics and reproductive capacity, but also during fetal development. Finally, the agreement between the chromosomal, gonadal, internal genital, external genital, and endocrinological sex may be impaired if the individuals do not identify psychologically with the sex in which they are classified (psychological sex); additionally, the social acceptance of these individuals in one sex or another should be considered (social sex).^1^, ^2^, ^3^

When dealing with a patient with DSD, especially children with genital ambiguity, the main objective is to make an accurate etiological diagnosis. This diagnosis will lead to a correct definition of the sex, timing, and type of reconstructive surgical correction of the external genitalia, prediction of the development of spontaneous secondary sexual characteristics, need for future hormone replacement therapy, estimation of the risk of gonadal malignancy and appropriate time for gonadectomy (if necessary), and the possibility of future fertility. In many cases, genetic counseling for the family also depends on the etiological diagnosis, as well as for the individual themselves in cases where fertility is preserved.^1^, ^2^, ^3^

In cases of DSD with genital ambiguity, an incorrect diagnosis may lead to a future inconsistency between the individualʼs social and psychological sex. Therefore, the identification of a DSD case includes not only a prompt investigation but also an agile and rapid assessment. A precise diagnosis minimizes the psychological and social challenges faced by the family, alleviating the uncertainty about the sex of their child.^1^, ^2^, ^3^

As of September 2021, in Brazil, the Ministry of Health implemented a regulation stating that in instances where there is uncertainty about sex at birth, children are to be registered as “ignored sex”. This designation is subject to correction only after a comprehensive diagnostic investigation, ensuring the child's access to essential consultations and examinations through the Unified Health System (SUS) or any affiliated agreement.^4^^,^^5^

Due to their diagnostic and therapeutic complexity, patients with DSD require a trained multi-professional team and an arsenal of subsidiary tests to define the etiology and the sex of rearing as early as possible.^1^, ^2^, ^3^ The pediatrician, especially the neonatologist, is a key member of this team, as he or she is responsible for the early identification of genital ambiguity, first contact with the family, and appropriate referral to the multi-professional team.^6^

From January 1989 to December 2021, the team composed of a pediatrician, endocrinologist, geneticist, pediatric surgeon, psychologist, and social worker has seen approximately 2000 patients and identified a DSD etiology in more than half of them. Approximately 50 % of the patients were evaluated due to genital ambiguity.

In the literature, there is no large-scale publication from a single center reporting the association between the clinical and laboratory features of children with genital ambiguity and their defined sex of rearing. Therefore, based on this uniform experience and large sample, the aim of this study was to verify the relationship between the clinical and cytogenetic characteristics of these patients and their defined sex of rearing.

## Methods

The sample comprised all DSD patients who came to this service from January 1989 to December 2021 due to genital ambiguity,^1^^,^^2^ who had confirmed etiological diagnosis, and who had not a sex assignment prior to the first consultation.

The data were obtained from a survey of medical records of the University Hospital. The following data were collected: Year of birth (grouped into ≤ 1999, 2000 to 2006 and > 2006); year of first consultation (grouped into ≤ 1999, 2000 to 2006 and > 2006); age at first consultation (grouped as 0–1 month and 2–12 months); severity of genital ambiguity according to Prader scale^7^ (grouped as 1 to 2, 3 and 4 to 5); the presence of palpable gonad(s) (grouped as yes and no); the presence of uterus on ultrasound (grouped as yes and no); karyotype (grouped as sex chromosome abnormalities, 46,XY and 46,XX); confirmed etiological diagnosis (grouped as disorder of gonadal determination, 46,XY testicular and 46,XX ovarian; the gender identity and sexual orientation in patients over 18 years of age at last consultation (according to auto-declaration in medical records); and the calculated age at December 2023.

There were no exclusion criteria for this study, except when the medical records could not be found or when the necessary information was incomplete. As this study involved only a review of medical records, a waiver of the informed consent form was requested and approved by the Research Ethics Committee of the University (CAAE: 97392018.0.0000.5404). This was a retrospective study, involved a survey of data already present in the patient's records, and it did not involve any risk to them. The identity (name) of the patients will not be disclosed at any time during the study.

Statistical analysis was carried out using SPSS (*Statistical Package for the Social Sciences*, Inc., Chicago, IL, USA) version 20.0. The data are presented in tables. An initial analysis was made of the dependent variable (sex of rearing) in relation to the independent variables (year of birth, year of consultation, age, Prader, presence of uterus on ultrasound, karyotype, and final diagnosis) using the chi-square test, with a significance level of less than 0.05. The adjusted *Odds Ratio* values were estimated using multivariate logistic regression, *Forward Stepwise* (*Wald*) method with a probability of inclusion of 0.05 and exclusion of 0.10.

## Results

One hundred and thirty-three cases were included, of which 74 were reared as males and 59 as females. All data were available in the medical records. Table 1 shows the association of the independent variables analyzed in relation to the defined sex of rearing. There was no association of year of birth and year of first consultation with the definition of sex of rearing, but there was a significant association of age at first consultation, degree of genital ambiguity using 1the Prader scale, presence of palpable gonad(s), presence of uterus on ultrasound, karyotype, and diagnosis with the definition of sex of rearing (Table 1).

**Table 1:** Association of the defined sex of rearing of 133 cases of genital ambiguity, with clinical, ultrasound and cytogenetic variables.

		Sex of Rearing				
		Male	Female	DF	χ^2^	*p*
Year of Birth	≤ 1999	26	26	2	1.325	0.516
	2000 a 2006	20	12			
	> 2006	28	21			
Year of Consultation	≤ 1999	26	26	2	1.420	0.492
	2000 a 2006	19	11			
	> 2006	29	22			
Age at first appointment	0 to 1 month	46	28	1	8.338	0.006
	2 to 12 months	50	9			
Prader	1 a 2	2	18	2	21.809	0.0001
	3	32	24			
	4 a 5	40	17			
Palpable gonad(s)	Yes	68	16	1	59.190	0.0001
	No	6	43			
Presence of a uterus	Yes	15	52	1	60.480	0.0001
	No	59	7			
Karyotype	SCA	6	8	2	86.195	0.0001
	46,XY	67	8			
	46,XX	1	43			
Diagnostics	DGD	21	11	2	78.701	0.0001
	46,XY testicular	53	7			
	46,XX ovarian	0	41			

SCA, Sex Chromosomes Abnormalities; DGD, Disorder of Gonadal Development;

DF, Degrees of Freedom; χ^2^, chi-square.

For the multivariate logistic regression analysis, three models were developed by considering the male gender (Table 2).

**Table 2:** Models proposed using multivariate logistic regression analysis to define the male sex of 133 cases of genital ambiguity according to clinical, ultrasound and cytogenetic variables.

Model 1: Clinical assessment only				
		*p*	OR	95 %CI
Palpable gonad(s)	Yes	0.001	25.70	9.01–73.32
	No	–	1.00	
Prader Group	4 a 5	0.015	9.66	1.54–60.56
	3	0.041	6.68	1.08–41.39
	1 a 2	–	1.00	
Model 2: Clinical assessment with ultrasound				
Palpable gonad(s)	Yes	0.001	14.19	3.85–52.36
	No	–	1.00	
Prader Group	4 a 5	0.002	39.15	3.70–413.75
	3	0.007	23.59	2.39–232.57
	1 a 2	–	–	
Presence of a uterus	No	0.001	24.95	6.64–93.80
	Yes	–	–	
Model 3: Clinical assessment with ultrasound and karyotype				
Palpable gonad(s)	Yes	0.966	–	–
	No	–	–	–
Prader Group	4 a 5	0.007	28.14	2.50–316.78
	3	0.009	25.17	2.24–282.43
	1 a 2	–	1.00	–
Presence of a uterus	No	0.014	8.22	1.53–44.29
	Yes	–	1.00	–
Karyotype Group	46,XY	0.001	123.80	12.39–1237.44
	SCA	0.006	26.11	2.49–273.33
	46,XX	–	–	–

SCA, Sex Chromosome Abnormalities; OR, Odds Ratio; CI, Confidence Interval.

In model 1 (only clinical assessment), the chance of a child with genital ambiguity being assigned as male (instead of female) was 25.7 times higher when at least one of the gonads was palpable; 9.66 times higher if the severity of the genital ambiguity was Prader group 4 or 5; and 6.68 times greater if it Prader group was 3.

In model 2 (clinical assessment combined with ultrasound), the chance of a child with genital ambiguity being assigned as male (instead of female) was 14.9 times higher when at least one of the gonads was palpable; 39.15 times higher if the severity of the genital ambiguity was in Praderʼs group 4 or 5; 23.59 times higher if Prader group was 3; and 24.95 times higher if there was no uterus on ultrasound.

In model 3 (clinical assessment combined with ultrasound and karyotype), the chance of a child with genital being assigned as male (instead of female) was 28.14 times higher if the severity of genital ambiguity was Prader group 4 or 5; 25.17 times higher if Prader group was 3; 8.22 times higher if there was no uterus on ultrasound; 123.80 times higher if the karyotype was 46,XY; and 26.11 if it was a sex chromosome abnormality. In this model, the palpable gonad(s) variable was not a predictor of male sex.

In December 2023, from 133 patients, 77 (58 %) were over 18 years old; 19 (14 %) had between 14 and 17 years, 188 (13 %) had between 10 and 13 years, and 19 (14 %) less than 10 years. All adult patients had a gender identity compatible with the sex of rearing, though 9 of 77 (12 %) had a homo or bisexual orientation, including 7 women with CAH and 2 men with idiopathic testicular 46,XY DSD. No adult patients requested gender reassignment. Supplementary data (supplement) show all etiological diagnoses grouped by sex of rearing.

## Discussion

The birth of a child with DSD is not always a medical emergency. However, a newborn with ambiguous genitalia is disconcerting, alarming, and considered a social emergency. Everyone is interested in knowing if the baby is a boy or a girl. Until the sex of rearing is established, the child should be referred to as “your baby”^8^ and the child registered as sex “ignored sex”.^4^^,^^5^ A comprehensive clinical, cytogenetic, hormonal, imaging and molecular assessment should be carried out, before sex assignment and this information shared with the parents. The tone of this meeting should be positive and optimistic to promote the connection between parents and baby, in addition to the link between family and physician. During this conversation, the sex of rearing, medical management plans, gonadal development, genetic test results, recurrence risks, and follow-up plans should be discussed.^8^

The decision on the sex of rearing is crucial for the individualʼs future and considers several factors, including cultural and religious background, future fertility, degree of virilization, potential for adult sexual function, need of surgery, lifelong replacement therapy, and risk of gonadal neoplasia.^1^, ^2^, ^3^ However, the main parameter to be considered is the likely gender identity in adulthood, which is strongly dependent on the etiology of DSD.^9^ As the decision on the sex of rearing is very important for the future prognosis of the child with DSD, the identification of relatively simple clinical, ultrasound, and cytogenetic issues may help reach a quick and reliable decision.

The definition of sex of rearing has undergone changes over the years, having gone through an exclusively surgical era (until the 1990s) where techniques for reconstructing female genitalia were much better known and performed, and therefore the choice of the female sex predominated,^10^ passing through a period where there were questions about neutral sexual identity up to the age of two, especially after the publication of the *John versus Joane* case in the 1990s,^10^^,^^11^ and culminating in the DSD consensus published in 2006^1^ and revised in 2016.^2^

There is evidence that the definition of the sex of rearing and acceptance of sexuality differs significantly between various societies and cultures, whether due to social, cultural, or religious aspects.^12^ In most societies, the social and economic position of men differs significantly from that of women, and the male sex seems to offer more and better life options in these cases. The doctorʼs own cultural background may also influence the decision on the sex of the child, which reinforces the importance of the interdisciplinary team.^1^, ^2^, ^3^ Therefore, in the discussion with the family to decide on the sex of the child, one should not fail to consider social, cultural, ethnic, and religious aspects specific to that family or the society in which it operates.^1^, ^2^, ^3^^,^^13^, ^14^, ^15^, ^16^, ^17^

This study showed that there was no association between year of birth and year of first consultation with the definition of sex of rearing, but there was an association between age at first consultation, degree of genital ambiguity using the Prader scale, presence of palpable gonad(s), presence of uterus on ultrasound, karyotype, and diagnosis with the definition of sex of rearing. That is, easily obtainable data can assist in determining sex; however, the discussion with parents and a multi-professional team is essential.

This study also showed that just assessing the severity of genital ambiguity and the presence of palpable gonads may increase the chance of defining the male sex by up to 25 times. When ultrasound is added, this chance may increase 40 times. When karyotyping is combined, it may be increased by more than 120 times. In this group, the data of the patients in adulthood with the gender identity in accordance with their sex of rearing and without cases of gender reassignment show the importance of having criteria for defining the sex of rearing and the need for the presence of a multi-professional team in the diagnosis and long-term follow-up of these cases.

Therefore, it is concluded that clinical, ultrasound, and simple cytogenetic data are strongly associated with the definition of the sex of rearing of children with genital ambiguity and without a defined sex at the first evaluation. Furthermore, this study indicates that a muti-professional approach allows a definition of sex assignment in newborns which will be appropriate for the gender identity and sexual orientation of these individuals in adulthood.

## Conflicts of interest

The authors declare no conflicts of interest.

## Appendix Supplementary materials

Image, application 1
