# Supplementary material for: Patients with genital ambiguity referred without a sex definition: the relationship between clinical picture and defined sex of rearing
Source: J Pediatr (Rio J). 2024 May 29;100(6):609–13. doi: 10.1016/j.jped.2024.05.001 (PMC11662739; doi:10.1016/j.jped.2024.05.001)
Supplement: Supplementary file 1 [file mmc1.docx]

**JPED-D-23-00529 – Supplementary Material**

**Supplement**

Etiological diagnoses of 133 cases of DSD with genital ambiguity evaluated before the definition the sex of rearing and the final sex of rearing.

| **Group** | **Etiological Diagnosis** | **n** | **Male** | **Female** |
| --- | --- | --- | --- | --- |
| Gonadal Determination Disorder | - XY Partial Gonadal Dysgenesis | 15 | 15 | 0 |
|  | - Mixed Gonadal Dysgenesis | 14 | 6 | 8 |
|  | - Ovotesticular DSD | 3 | 0 | 3 |
| 46,XY testicular DSD | - PAIS | 5 | 2 | 3 |
|  | - 5α-reductase deficiency type 2 | 5 | 5 | 0 |
|  | - Syndromic | 12 | 12 | 0 |
|  | - Idiopathic | 38 | 34 | 4 |
| 46,XX ovarian DSD | - CAH – 21α-OHase deficiency | 33 | 0 | 33 |
|  | - CAH – 3β-OH-steroid deficiency | 2 | 0 | 2 |
|  | - CAH – 11β-OHase deficiency | 1 | 0 | 1 |
|  | - Syndromic | 5 | 0 | 5 |

CAH = Congenital Adrenal Hyperplasia; DSD = Disorder of Sex Development; PAIS = Partial Androgen Insensitivity Syndrome
